# Potential role for immune-related genes in autism spectrum disorders: Evidence from genome-wide association meta-analysis of autistic traits

**DOI:** 10.1177/13623613211019547

**Published:** 2021-08-04

**Authors:** Martina Arenella, Gemma Cadby, Ward De Witte, Rachel M Jones, Andrew JO Whitehouse, Eric K Moses, Alex Fornito, Mark A Bellgrove, Ziarih Hawi, Beth Johnson, Jeggan Tiego, Jan K Buitelaar, Lambertus A Kiemeney, Geert Poelmans, Janita Bralten

**Affiliations:** 1 Institute of Psychiatry, Psychology and Neuroscience, King’s College London, UK; 2 Radboud University Medical Center, The Netherlands; 3 The University of Western Australia, Australia; 4 University of Tasmania, Australia; 5 Turner Institute of Brain and Mental Health, Australia; 6 Monash University, Australia; 7 Donders Institute for Brain, Cognition and Behaviour, The Netherlands; 8 Karakter Child and Adolescent Psychiatry University Centre, The Netherlands

**Keywords:** autism spectrum disorders, genetics, immune system, molecular and cellular biology

## Abstract

The clinical heterogeneity of autism spectrum disorders majorly challenges their genetic study. Autism spectrum disorders symptoms occur in milder forms in the general population, as autistic-like traits, and share genetic factors with autism spectrum disorders. Here, we investigate the genetics of individual autistic-like traits to improve our understanding of autism spectrum disorders. We meta-analysed four population-based genome-wide association studies investigating four autistic-like traits – ‘attention-to-detail’, ‘imagination’, ‘rigidity’ and ‘social-skills’ (*n* = 4600). Using autism spectrum disorder summary statistics from the Psychiatric Genomic Consortium (*N* = 46,350), we applied polygenic risk score analyses to understand the genetic relationship between autism spectrum disorders and autistic-like traits. Using MAGMA, we performed gene-based and gene co-expression network analyses to delineate involved genes and pathways. We identified two novel genome-wide significant loci – rs6125844 and rs3731197 – associated with ‘attention-to-detail’. We demonstrated shared genetic aetiology between autism spectrum disorders and ‘rigidity’. Analysing top variants and genes, we demonstrated a role of the immune-related genes *RNF114, CDKN2A, KAZN, SPATA2* and *ZNF816A* in autistic-like traits. Brain-based genetic expression analyses further linked autistic-like traits to genes involved in immune functioning, and neuronal and synaptic signalling. Overall, our findings highlight the potential of the autistic-like trait–based approach to address the challenges of genetic research in autism spectrum disorders. We provide novel insights showing a potential role of the immune system in specific autism spectrum disorder dimensions.

**Lay abstract:**

Autism spectrum disorders are complex, with a strong genetic basis. Genetic research in autism spectrum disorders is limited by the fact that these disorders are largely heterogeneous so that patients are variable in their clinical presentations. To address this limitation, we investigated the genetics of individual dimensions of the autism spectrum disorder phenotypes, or autistic-like traits. These autistic-like traits are continuous variations in autistic behaviours that occur in the general population. Therefore, we meta-analysed data from four different population cohorts in which autistic-like traits were measured. We performed a set of genetic analyses to identify common variants for autistic-like traits, understand how these variants related to autism spectrum disorders, and how they contribute to neurobiological processes. Our results showed genetic associations with specific autistic-like traits and a link to the immune system. We offer an example of the potential to use a dimensional approach when dealing with heterogeneous, complex disorder like autism spectrum disorder. Decomposing the complex autism spectrum disorder phenotype in its core features can inform on the specific biology of these features which is likely to account to clinical variability in patients.

## Introduction

Autism spectrum disorders (ASDs) refer to a class of common and pervasive conditions with an early life onset (Lai et al., 2014). Core ASD characteristics are impaired social communication and interaction, and repetitive, restrictive interests and behaviours, along with sensory abnormalities (Hazen et al., 2014). These symptoms impact on patients’ quality of life and on individual caretakers and society (Billstedt et al., 2011). Considering the increasing prevalence of ASDs (~2%) and the lack of effective treatments, there is an imperative to understand ASD aetiology (Fombonne, 2018; Kim et al., 2011).

Twin studies indicate that ASDs are highly heritable (h^2^~70%–90%), demonstrating the importance of genetic research on these conditions (Tick et al., 2016). However, ASDs are genetically complex and multifactorial, with rare and common variants involved (de la Torre-Ubieta et al., 2016; Grove et al., 2019). Genome-wide association studies (GWASs), comparing cases to controls, represent the gold standard for identifying common genetic risk variants for multifactorial disorders like ASDs. To date, GWASs have been limited as extremely large samples are needed to find robustly associated risk variants. The most recent ASD-GWAS meta-analysis included 18,381 cases and 27,969 controls and detected five independent genome-wide significant loci (Grove et al., 2019). Two additional genome-wide significant loci were identified after meta-analysing these data with genetic data from the European cohort of the Simons Foundation Powering Autism Research Knowledge (SPARK) project, leading to a total sample size of 55,420 (Matoba et al., 2020). Functional analysis of the ASD-GWAS top findings, together with rare genetic variant and animal studies, revealed a broad molecular landscape for ASD, involving steroidogenesis, and neurobiological processes, like neurite outgrowth and synaptic function (Poelmans et al., 2013). The neurobiological nature of ASDs is confirmed by neuroimaging studies reporting neuroanatomical alterations in ASDs, although results differ across individuals (Chen et al., 2019; Van Rooij et al., 2018). Nevertheless, the largest to-date meta-analysis showed robust differences in the frontal and striatal regions in ASD cases compared to controls (Van Rooij et al., 2018).

Overall, phenotypic heterogeneity constitutes a major obstacle to the study of ASD aetiology, and, therefore, it is important to address this issue. It has been demonstrated that complex disorders like ASDs represent the extreme manifestation of quantitative traits occurring in the general population along a continuum (Constantino & Todd, 2003). Autistic-like traits (ALTs) refer to those continuous variations in social skills and repetitive behaviours that in their most severe forms define ASDs (Constantino & Todd, 2003). Each ALT captures a distinct ASD feature, parsing the complex autistic phenotype. The continuous ALT distribution is determined by the cumulative effect of many common, small-effect genetic variants that collectively increase ASD risk (Weiner, 2017). Hence, the genetic study of ALTs may constitute a route to disentangle the complex ASD genetics. Previous studies highlighted the potential of ALT-based research to investigate ASD aetiology (Colvert et al., 2015; Constantino & Todd, 2003; Jones, 2015; Lundström et al., 2012; Robinson et al., 2013, 2016; Taylor et al., 2019). These studies, in fact, demonstrated genetic correlations between ASDs and social and communication skills in the general population. In addition, Bralten et al. (2018) defined five core ALTs – attention-to-detail, childhood behaviour, imagination, rigidity and social skills – through factor analyses of ASD measurements, and indicated a shared genetic aetiology between specific traits and clinical ASDs.

The GWAS approach has gained popularity in studying population-based, quantitative traits (McCarthy et al., 2008). For ALTs, GWASs revealed suggestive associated genomic loci (Jones, 2015). However, previous ALT-GWASs in unrelated individuals from the general population relied on limited samples (*N* = ~2000), lacking power to detect robust genome-wide associations (Bralten et al., 2018; Robinson et al., 2016). Future research on larger study populations is therefore needed to detect ALT-associated variants. Moreover, ALT-GWASs thus far investigated social skills, whereas rigidity and attention have been under-represented (Jones, 2015). Considering the ASD phenotypic diversity, research encompassing a wide range of ALTs is needed. Well-powered, comprehensive ALT research may lead to novel genetic findings and reveal biological pathways involved in the ALT-ASD continuum.

This study investigates the genetics and biology of four ALTs: ‘attention-to-detail’, ‘imagination’, ‘rigidity’ and ‘social-skills’. First, we aimed to identify common genetic risk variants associated with ALTs. Therefore, we meta-analysed GWAS data for the ALTs from four cohorts of in total 4600 individuals and we assessed the shared genetic aetiology between ALTs and ASDs. Subsequently, we identified ALT-associated genes through gene-wide analyses that we combined with gene-expression network analyses to identify biological pathways associated with ALTs across established ASD-related brain regions.

## Methods

### Study cohorts and consortia

This study is an international collaboration including raw genotyping data and GWAS summary statistics from four study cohorts (Table 1) Community members were not included in this study.

**Table 1.** Descriptive statistics of the four population cohorts included in the meta-analysis.

Cohort	*N*	Mean age (SD)	Gender (% male)	Genotyping platform	Mean ALT scores (SD)			
					Attention	Imagination	Rigidity	Social skills
NBS	2847	28.4 (2.7)	46%	Illumina Human OmniExpress BeadChip	6.4 (1.2)	5.4 (1.7)	8.4 (2.6)	6.3 (2.1)
BIG	372	25.6 (4.5)	43%	Affymetrix GeneChip Array 6.0	6.3 (1.4)	4.3 (1.4)	8.3 (2.1)	6.11 (1.8)
Genetics of Cognition	416	24.4 (4.7)	39%	Illumina Infinium PsychArray-24 BeadChip	5.8 (2.1)	4.6 (1.78)	7.6 (1.6)	5.6 (2.2)
Raine	965	19.7 (0.7)	49%	Illumina Human 660W Quad array	5.4 (1.5)	4.0 (1.4)	9.7 (1.7)	7.6 (1.4)

SD: standard deviation; ALT: autistic-like trait; NBS: Nijmegen Biomedical Study; BIG: Brain Imaging Genetics.

Information about sample size, age, gender and genotyping platform for the four cohorts included (Franke et al., 2010; Galesloot et al., 2017; Jones et al., 2013; Pinar et al., 2018).

#### Nijmegen Biomedical Study

The Nijmegen Biomedical Study (NBS; http://www.nijmegenbiomedischestudie.nl/) is a population-based study set up by the Department of Health Evidence and the Department of Laboratory Medicine of the Radboud University Medical Center (Radboudumc) in Nijmegen, The Netherlands. The NBS investigates the role of genetic and environmental factors on individual well-being (Galesloot et al., 2017). The study was approved by the Institutional Review Board of Radboudumc (CMO 2001/055). All participants completed written informed consent. This study included imputed genotyping data and ALT scores for a total of 2847 Dutch individuals participating in the NBS.

#### Brain Imaging Genetics

The Brain Imaging Genetics (BIG; http://www.cognomics.nl/big.html) project is an initiative promoted by the Human Genetics Department of the Radboudumc, the Donders Centre for Cognitive Neuroimaging of the Radboud University, and the Max Planck Institute for Psycholinguistics in Nijmegen, The Netherlands. The BIG project investigates genetic variation linked to behaviour, cognition, brain structure and function in the general population (Franke et al., 2010). The BIG study was approved by the regional medical ethics committee (CMO Regio Arnhem/Nijmegen). All participants provided written informed consent. This study included imputed genotyping data and ALT scores for a total of 372 individuals of European ancestry participating in the BIG project.

#### The Raine Study

The Raine Study (https://rainestudy.org.au/) is a large prospective cohort study of pregnancy, childhood, adolescence and adulthood based in Western Australia. The study investigates the role of genetic and environmental factors on individual well-being using a longitudinal approach (Jones et al., 2013). The Raine Study was approved by the Human Research Ethics Committee at the King Edward Memorial Hospital and University of Western Australia. The Raine Study Gen2 participants and their family provided written informed consent. This study used GWAS summary statistics of four ALTs for a total of 945 European individuals participating in the Raine Gen2-20 year follow-up study.

#### Genetics of Cognition

Genetics of Cognition (GenofCog) is a general population study set up by the Turner Institute of Brain and Mental Health and the Monash University in Melbourne, Australia. The study investigates genetic variations and neural correlates related to cognition and psychopathology (Pinar et al., 2018). The study was approved by the Monash University Ethics Committee. All participants provided written informed consent. This study used imputed genotyping data and ALTs score for 436 European individuals participating in the GenofCog study.

### Assessment of autistic traits

The Autism Spectrum Quotient (AQ) (Baron-Cohen et al., 2001) and a customised ALT questionnaire developed by Bralten et al. (2018) were used to measure the four ALTs across our cohorts. The AQ, a self-report 50-item questionnaire, was adopted to assess ALTs in the Raine Study and in the GenofCog cohorts. The customised ALT questionnaire is a self-report, shorter measure consisting of 18 items of which 12 are AQ-derived and six refer to ASD criteria described in the Diagnostic and Statistical Manual of Mental Disorder (Bralten et al., 2018). The questionnaire demonstrated construct validity, as shown by a moderately high internal consistency (Cronbach’s α = 0.70) of the total score to the 18 items (Bralten et al., 2018). Bralten et al. (2018) showed that the 12 AQ-derived items of this questionnaire converge on four factors – ‘attention-to-detail’, ‘imagination’, ‘rigidity’ and ‘social-skills’ – while the six Diagnostic and Statistical Manual of Mental Disorders (DSM)-based items cluster into a fifth factor: ‘childhood-behaviour’(Bralten et al., 2018). These items explain 50.7% of the variance in the total autistic score. This questionnaire was adopted to measure ALTs in the NBS and BIG samples. To ensure homogeneity across studies, we exclusively considered the 12 items shared between the two questionnaires and therefore investigated the ALTs ‘attention-to-detail’, ‘imagination’, ‘rigidity’ and ‘social-skills’. A list of the 12 items considered for this study can be found in Supplementary Table 2. Individual ALT scores were calculated, and outliers (2 standard deviations (2SD) from the mean) removed (see Table 1 for mean ALT values). Scores were then corrected for age and sex and residuals were used in following analyses after being checked for independence and log-transformed to ensure normality using SPSS21.

### Genome-wide association analyses and meta-analyses

Genotyping was performed on study-specific platforms (Table 1). Initial single nucleotide polymorphism (SNP) filtering was applied on call rate (>95%), Hardy–Weinberg equilibrium (HWE <1^−6^) and minor allele frequency (MAF >0.01). In each cohort, genotyped data for the autosomal chromosomes were imputed to increase genotype density and achieve fine genome-wide mapping. For all data sets, imputation followed the ENIGMA protocol (http://enigma.ini.usc.edu/wp-content/uploads/2012/07/ENIGMA2_1KGP_cookbook_v3.pdf), that adopts the 1000 Human Genome Project reference panel and the software MACH (Li et al., 2010). SNPs with imputation quality <0.7 were excluded. Multidimensional scaling (MDS) was performed to assess population structure. Next, independent ALT-GWASs were performed in each cohort implementing a linear regression model in Mach2qtl, fitting the quantitative nature of ALTs. The model included sex, age and the four MDS components as covariates. Cohort-specific results were quality-controlled using the *clean* function of the *EASYQC* package in R (Winkler et al., 2014) and data were combined in four ALT-specific inverse-variance weighted meta-analyses in METAL (Willer et al., 2010). The final analysis included a total of 8,284,544 autosomal SNPs. We applied the significant *p*-threshold of *p* < 1.25^−9^, referring to the canonical GWAS *p*-threshold (*p* < 5^−8^) divided by the ALTs tested. In order to estimate the statistical power we had to find genome-wide significantly associated SNPs in our meta-analysis of 4600 individuals, we performed a power calculation in QUANTO (Gauderman, 2002). The power analysis showed that GWAS of quantitative traits, under the assumption of normal trait distribution, required more than 4000 individuals to identify SNPs with frequency >1% at 80% power.

### Shared genetic aetiology analysis

Polygenic risk score (PRS)–based analyses were applied to estimate the extent of shared, common variant, genetic aetiology between clinical ASDs and ALTs using PRSice (v1.25) (Euesden et al., 2015). To set the ‘base ASD phenotype’ we used publicly available ASD-GWAS summary statistics by an independent cohort, not-overlapping with the ALT cohorts from the Psychiatric Genomic Consortium (PGC) and the Lundbeck Foundation Initiative for Integrative Psychiatric Research (iPSYCH) counting 18,381 cases and 27,969 controls (The Autism Spectrum Disorders Working Group of The Psychiatric Genomics Consortium, 2017). Summary statistics for ALTs (obtained from the meta-analysis of our cohort-specific GWAS data) were used to define the ‘target’ phenotypes. Four separate PRS-based analyses have been conducted between ASDs and each ALT at a time, using the summary–summary statistics-based approach (Euesden et al., 2015). Clumping, using PLINK, preceded the actual PRS calculation to ensure that only index SNPs for each linkage disequilibrium (LD) block (*r*^2^ < 0.25, 500 kb) throughout the genome were considered. Next, PRSs were calculated on the clumped ASD summary statistics and included only SNPs exceeding seven broad *p*-value thresholds (i.e. *P_T_* < 0.001, 0.05, 0.1, 0.2, 0.3, 0.4 and 0.5). For each threshold, ASD-PRS was extracted and used to estimate the extent of shared genetic aetiology with ALTs. *p-*values were corrected using the False Discovery Rate (FDR) method in R.

### Gene-wide analyses

Gene-wide analyses on ALT-GWAS results were performed to identify ALT-associated genes using the Multi-marker Analysis of GenoMic Annotation (MAGMA) software (de Leeuw et al., 2015). First, ALT-SNPs were annotated to genes using 100 kb downstream and upstream flanking regions to include regulatory regions. Next, a gene-specific *Z*-statistic was obtained considering the *p*-values of gene-related SNPs, while correcting for LD. We applied the significance *p*-value threshold *p* = 2.8^-6^, accounting for the total number of genes tested.

### Gene co-expression network analyses

To explore the functional role of ALT-associated SNPs, we performed gene co-expression network analysis using the eQTL-MAGMA (e-MAGMA) software package (Gerring et al., 2019). Analyses followed the procedure described in https://github.com/eskederks/eMAGMA-tutorial. Accordingly, we mapped SNPs to genes based on available annotation files, that were tissue-specific and referred to significant (FDR <0.05) SNP-gene associations from GTEx (https://www.gtexportal.org/home/). Next, we performed gene-wide analyses to link ALT-GWAS SNPs to eQTL-associated gene (eGenes) using annotation files for seven ASD-associated brain regions (i.e. total cortex, frontal cortex, anterior cingulate cortex, putamen, caudate, nucleus accumbens and amygdala) (Van Rooij et al., 2018). Gene-wide analyses adopted the MAGMA approach and provided an eGene-specific *Z*-statistic reflecting association with ALTs. Subsequently, we performed gene-set expression analyses using gene-set annotations referring to region-specific co-expression gene-networks. These region-specific co-expression networks are divided into sets or modules (indexed by colour) of correlated genes. Using the MAGMA gene-set approach, we performed a competitive test testing the association of each module with the ALTs. Results were then Bonferroni-corrected (i.e. accounting for the total of gene-modules tested). Finally, we performed post hoc analyses on the significant gene-set associations to define the biological functions of the identified gene-expression modules. We used the g:GOst tool from the g:Profiler webserver (https://biit.cs.ut.ee/gprofiler/gost) (Kull et al., 2007), which performed gene-set enrichment analyses on input gene lists using a Fisher’s one-tailed test, based on Gene Ontology (GO) annotations (http://geneontology.org/). The g:SCS option was chosen to correct for multiple testing while controlling for the inter-correlation between GO terms.

We integrated brain-specific gene expression analyses for ALTs with gene expression analyses across a wider range of human tissues. To do so, we exploited the tool for functional mapping and annotation (FUMA) of GWAS that refers to tissue-specific expression patterns based on GTEx v6 RNA-seq data (Watanabe et al., 2017). We used the summary statistics for each ALT as input.

## Results

### Meta-analysis of genome-wide association with autistic traits

**Figure 1.**
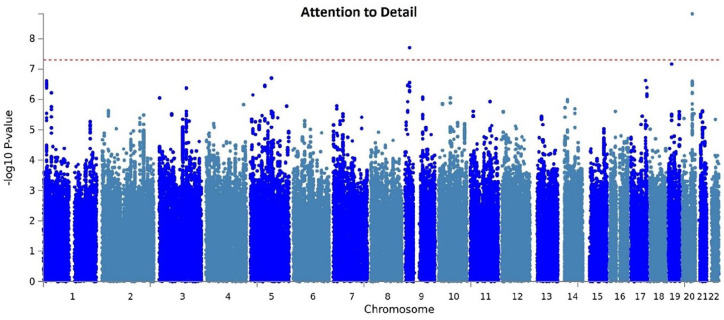
Manhattan plot of the GWAS meta-analysis for ‘attention to detail’. Each dot represents the result of the linear regression analysis for each single variant taking the attention to detail mean score as dependent variable and correcting for age, sex, gender and four MDS components. The *x*-axis shows the chromosomes and the *y*-axis shows the −log (two-sided) *p*-value of the association. The red dotted line indicates the threshold for genome-wide significance.

ALT-based meta-analyses revealed genome-wide significant associations of ‘attention-to-detail’ with two SNPs, *rs6125844* (*p* = 1.52 × 10^−^⁹) and *rs3731197* (*p* = 1.9 × 10^−^⁸) (Figure 1). The *rs6125844-*association survived our stringent correction (*p* < 1.25 × 10^−9^), whereas the *rs3731197-*association exceeded the genome-wide significance threshold (*p* < 5 × 10^−8^). No SNP-association reached significance for the other ALTs (*see Supplementary Information*).

### Shared genetic aetiology between ASDs and autistic traits

**Figure 2.**
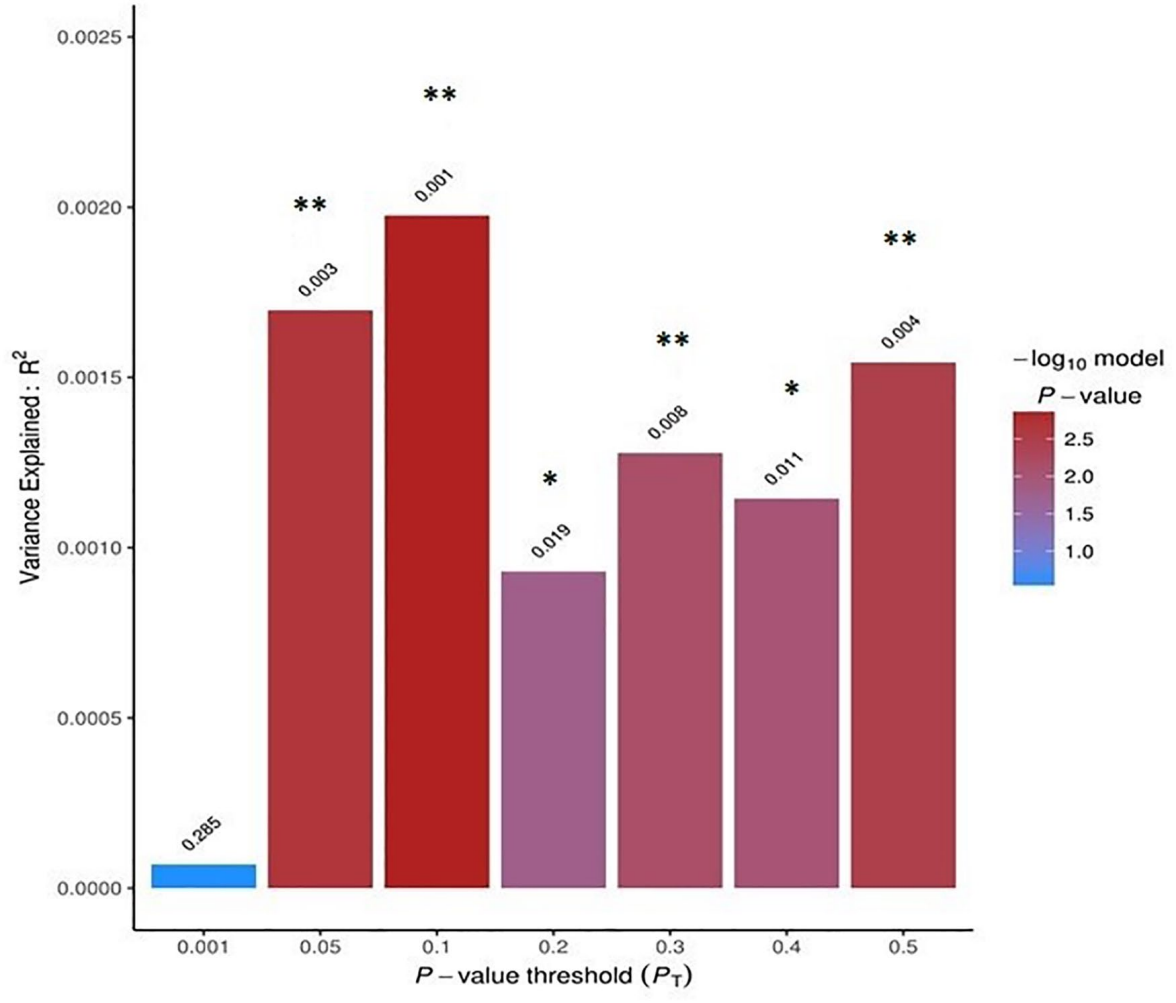
Polygenic risk-based results of ASDs and ‘rigidity’. Polygenic risk score-based results showing the degree of shared genetic aetiology between ASDs (‘base’ phenotype) and ‘rigidity’ (‘target’ phenotype) at seven broad *p*-value thresholds (*P_T_*). The bar plot was created with PRSice1. The *x*-axis displays the seven *p*-value thresholds tested and the *y*-axis displays the variance explained by the genetics of the ‘base’ phenotype in the ‘target’ phenotype. The colours of the bars indicated the −log10 *p*-value of the association. *p-values < 0.05 after FDR-correction; **p-values < 0.01 after FDR-correction.

Using the PRS-based approach, we found a shared genetic aetiology between clinical ASDs and ‘rigidity’ (Figure 2). The most predictive thresholds were *P_T_* = 0.05, *P_T_* = 0.1 and *P_T_* = 0.5 (FDR-corrected *p* < 0.01). Considering the common genetic variants captured by our analyses, we did not find a statistically significant genetic sharing between ASDs and other ALTs (see *Supplementary Information*).

### Gene-wide analyses

SNPs included in the ALT meta-analyses mapped onto a total of 17,867 autosomal genes. Gene-wide analyses showed that four genes – *RNF114, CDKN2A, SPATA2, KAZN –* were significantly associated with ‘attention-to-detail’. In addition, *ZNF816* was significantly associated with ‘social skills’ (Table 2). Literature-based analyses of these genes indicated an involvement in immune regulation and inflammatory phenotypes, like psoriasis. No gene-association survived the Bonferroni-correction for ‘imagination’ and ‘rigidity’.

**Table 2.** MAGMA-based significant results of gene-wide analyses.

ALT	Associated genes	*p*-value
*Attention to detail*	*RNF114* CDNK2 SPATA2 KAZN	2.05e−7 4.52e−7 2.30e−6 4.67e−7
*Imagination*	–	–
*Rigidity*	–	–
*Social skills*	*ZNF816A*	6.4e−7

MAGMA: Multi-marker Analysis of GenoMic Annotation; ALT: autistic-like trait.

Association results from MAGMA-based gene-wide analyses. Indicated top genes exceeded the Bonferroni-corrected threshold of *p* = 2.8^−6^ to account for the number of genes tested (*N* = 17,867).

### Gene co-expression network analyses

Gene co-expression network analyses revealed ALT-specific associations with gene-expression modules across ASD-related brain regions. Namely, we observed that attention-to-detail eGenes were statistically associated with the expression-module for total cortex (*p* = 0.001), while nominally associated with expression-module for putamen (*p* = 0.01). Imagination-related eGenes were associated the expression-module for nucleus accumbens (*p* = 0.003) and amygdala (*p* = 0.001). Rigidity-related eGenes were nominally associated with modules for anterior cingulate cortex (*p* = 0.01) and nucleus accumbens (*p* = 0.01). Finally, social skill–related eGenes were associated with an expression-module for anterior cingulate cortex (*p* = 0.001) and putamen (*p* = 0.002); functional enrichment analyses revealed a expression-module enrichment for biological processes, including synaptic signalling, neurogenesis. Among the enriched pathways we also identified immune-related processes, such as cytokine signalling, adding support to the results of our gene-wide analyses of ALTs. Results of the e-MAGMA and enrichment ALT-analyses are presented in the *Supplementary Information (Supplementary Table 1), as well as results from FUMA-based analyses of gene expression across multiple human tissues beyond brain (Supplementary Figures 8–11).* Additional data about GWASs results, PRS-based analyses and gene co-expression network analyses can be found in the *Supplementary Information* materials.

## Discussion

In this study, we investigated the genetics of four ALTs – ‘attention-to-detail’, ‘imagination’, ‘rigidity’ and ‘social-skills’ – by meta-analysing GWAS data of 4600 individuals from the general population. We found two common genetic variants (*rs6125844* and *rs3731197*) that were significantly associated with ‘attention-to-detail’. Our PRS-based analysis was significant for the comparison between ASDs and ‘rigidity’. Next, we showed significant associations between ‘attention-to-detail’ and *RNF114, CDKN2A, SPATA2* and *KAZN*, and between ‘social-skills’ and *ZNF816A*. Finally, we demonstrated that ALT-eQTLs are associated with gene-networks in ASD-related brain regions. Biological characterisation of these gene-networks showed enrichment in synaptic signalling, neurogenesis and the immune response.

By meta-analysing the largest available population-based data sets for ‘attention-to-detail’, ‘imagination’, ‘rigidity’ and ‘social-skills’, we were able to identify two SNPs, r*s6125844* and *rs3731197*, significantly associated with ‘attention-to-detail’. The SNP *rs6125844* is mapped to a *cis*-regulatory region for *RNF114*, potentially influencing its genetic expression. *RNF114* is an E3-ubiquitin ligase that has been implicated in immune reactivity through direct regulation of the NF-kB pathway and relation with innate immunity mediators, suggesting a role of immunity-related genetics in ‘attention-to-detail’ (Bijlmakers et al., 2011). Furthermore, *RNF114* promotes the ubiquitination and degradation of cyclin-dependent kinase inhibitors (CKIs), that contribute to neuronal functions, like axon guidance and synaptic signalling which have been linked to ASD pathophysiology (Kawauchi et al., 2013; Poelmans et al., 2013). These CKIs have also been proposed as putative targets for the resolution of ongoing inflammation (Laphanuwat & Jirawatnotai, 2019). In agreement with that, our second top-associated SNP, *rs3731197*, is located in an intron of *CDKN2A* and in a *cis-*regulatory region for *CDKN2B*, both belonging to the CKI complex. Both *CDKN2A* and *CDKN2B* encode key proteins important for neurodevelopment and show immune-regulatory properties (Kawauchi et al., 2013). In brief, our top SNP-associations suggest a link between ‘attention-to-detail’ and immune regulators, that deserves consideration in future ALT-based research.

Our findings confirm our hypothesis and previous results of a degree of genetic association between ALTs and ASDs (Robinson et al., 2016). The results of our PRS-based analyses, linking common variants for ASDs and ‘rigidity’, are in line with previous analyses on a subset of our data set that showed genetic sharing between ASDs and this ALT (Bralten et al., 2018). In general, these findings indicate an existing ALT-to-ASD genetic continuity and validate the idea of using ALT-data to address the complex genetics of ASDs. The quantitative-trait approach also fits with the research domain criteria (RDoC) paradigm promoted by the National Institute of Mental Health (NIMH) that aims to dismiss categorial classification of mental disorders in favour of dimensional definitions (Insel, 2014). By looking at specific functional domains, that cut across psychiatric categories, a quantitative-trait approach may circumvent the heterogeneity and comorbidity associated with DSM-based diagnoses. For instance, ‘rigidity’ is a trait observed in ASDs, obsessive-compulsive disorder (OCD) and anxiety disorders (Morris & Mansell, 2018). Research on rigidity may therefore offer insights into molecular mechanism(s) underlying all these conditions and ideally defining common target(s) of intervention.

Following GWAS meta-analyses, gene-wide analyses revealed that four genes – *RNF114, CDKN2A, SPATA2* and *KAZN* – were significantly associated with ‘attention-to-detail’. As mentioned, *RNF114* and *CDKN2A* encode proteins directly involved in immune regulation through their action on the NF-kB signalling pathway (Bijlmakers et al., 2011; Pramanik et al., 2018). Moreover, *SPATA2* is also shown to regulate TNF-induced NF-kB signalling and appears specifically expressed in testis Sartori cells, an immune-privileged site and has been implicated in inflammation (Schlicher et al., 2017). Given the strong male prevalence in ASDs, *SPATA2* expression in the testis may reveal a sex-specific effect of this gene in immune regulation. This hypothesis should be addressed in future research by adopting a sex-stratified approach to ALTs. Besides, we also observed a significant association between ‘social-skills’ and *ZNF816*. Like *RNF114, ZNF816* encodes for a zinc-finger protein involved in immune processes, like NF-KB signalling, and implicated in autoimmune diseases as shown in previous studies (Kallionpää et al., 2014; Stuart et al., 2015). These results, therefore, further suggest a role of immunity-related genetics in ALTs. Given the genetic correspondence between ASDs and ALTs, these results stress the importance to further investigate the relationship between immunogenetics and ASDs. Immune dysregulation, either in the form of ongoing inflammation or autoimmunity, is prevalent in ASDs (McAllister, 2017). ASDs are, in fact, associated with family history of autoimmune diseases, like celiac disease and rheumatoid arthritis (Atladóttir et al., 2009; Ludvigsson et al., 2013), and either increased or decreased levels of inflammatory markers have been found in the blood of ASD individuals (Goines & Ashwood, 2013). However, immune dysregulation varies among patients and it is purported to be confined to a specific ASD-subgroup (Careaga, 2017). This, together with our finding of a trait-specific immune link, suggests that immunity might be associated specifically with certain autistic features. ALT-based research could, then, clarify the complex relationship between immunity and ASDs, by revealing ALT-related immunobiological mechanisms and pathways for dimension-specific pharmacotherapy. This research line is of particular relevance for ASDs given the high heterogeneity of these disorders, that suggests the improbability of a one-fit-all treatment, but the need for an intervention that is tailored on the patient’s characteristics. To this regard, PRS-based analyses could help to identify potential clinical subtypes, that would benefit from specific treatment(s).

**Figure 3.**
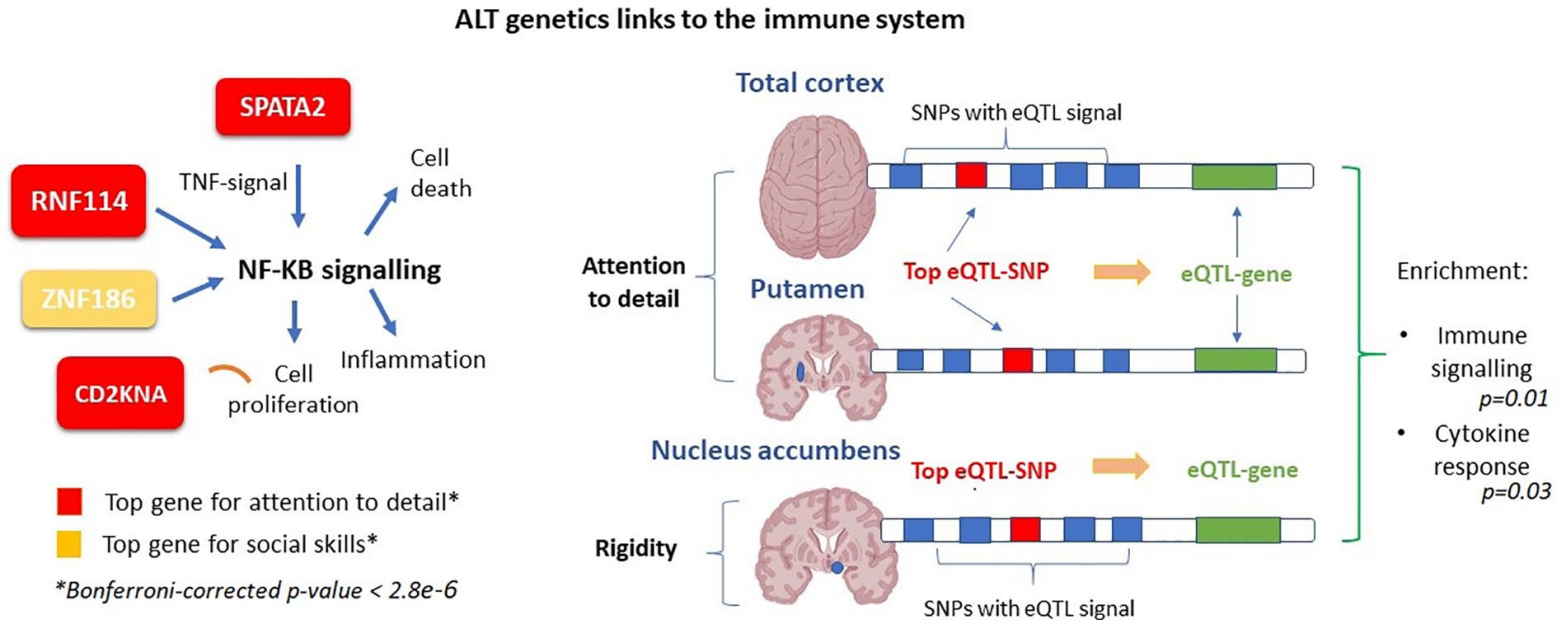
Summary figure of immune-related findings in ALT genetics. Summary figure to illustrate our findings pointing to a relationship between ALTs and the immune system. On the right, results of gene-wide analyses highlight significant ALTs associations with genes that are involved in immune functioning (NF-KB signalling). On the left, results of gene co-expression network analyses show that eQTL-genes, linked to ALTs, for total cortex, putamen and nucleus accumbens are enriched in immune processes (association *p*-values for the immune pathways are reported). *Genes exceeded the Bonferroni-corrected *p*-value threshold of 2.8e−6.

We followed our results with gene co-expression network analyses that assess the expression of genes derived by genetic variation in particular tissues, in our case established ASD-related brain regions. These analyses revealed an enrichment of neuronal and synaptic signalling in the cortex for ‘imagination’. These results are in line with previous literature showing that ASD-related genes are over-represented in neuronal processes and (glutamatergic) synaptic signalling (Poelmans et al., 2013). However, the fact that we observed trait-specific associations demonstrate biological variability between ALTs. This is supported by evidence from Warrier et al. (2019) of dissociable genetics between the ASD-like empathising and systematising behaviours. Importantly, synaptic dysregulation also occur in OCD (Ting & Feng, 2008), that often co-exists with ASDs and with these, shares behavioural rigidity (Meiran et al., 2011). Synaptic functioning may therefore constitute a common mechanism, potentially influencing the cross-disorder phenotype rigidity. In addition, enrichment analyses showed that ALT-related gene-networks across brain regions are involved in the immune response. Namely, we found that attention-to-detail and rigidity-related genes for total cortex, putamen and nucleus accumbens were enriched in immunological processes. These results corroborate SNP and gene-level findings results of an immune link to ALTs. Figure 3 provides an overview of our findings pointing to a link between the immune system and ALT genetics. In general, the observed enrichment of ALT-genes in neuro-immune processes demonstrates that both the immune and nervous system contribute to ALTs. This fits the evidence of a neuro-immune cross-talk during neurodevelopment and the findings of immune-related molecules driving neuronal growth and communication (Debnath et al., 2018; Nutma et al., 2019). Since ASDs are linked to aberrant neurodevelopment, it is important to understand the role of immune-related molecules along the ALT-to-ASD continuum.

This study should be evaluated in the light of some strengths and limitations. First, trait-oriented research exploits population-based cohorts for which large data are accessible at little cost. Relying on population-based cohorts, we combined data from multiple sources worldwide to obtain a sample large enough to perform a well-powered genetic ALT-investigation. The resulting sample size was, indeed, increased with respect to previous GWASs of ALTs that counted ~2000 unrelated individuals. Second, the analysis of our top findings offered new insights into the biology of autistic-like behaviours, hinting to the immune response. This does not only indicate potential areas for future investigation, but it helps to clarify the molecular profile underlying diverse autistic dimensions. The aggregation of multiple cohorts, however, increased the variability of this study population. Differences in gender distribution exist in the cohorts used (see Table 1). Although gender differences are documented in population-based surveys (Nutma et al., 2019), this might influence the representativeness of study cohorts and, therefore, more gender-balanced samples should be investigated. Also, our population included individuals exposed to different cultural and geographical backgrounds. Such differences may have biased, for example, the individual interpretation of ALTs and ultimately, the self-reporting of these traits. We indeed referred to self-report measurement that is intrinsically limited by the respondents’ interpretation of the items. The increase of heterogeneity, resulting from the aggregation of multiple datasets, could, in fact, explain our failure to replicate previous findings of a significant association between ‘rigidity’ and the *MET* gene as observed in only the NBS cohort. Also, although the levels of ALTs in our cohort have been checked for normality, we could not exclude the possibility that any individual received a formal ASD diagnosis. To this regard, we believe that future ALT-based research, adopting an ASD diagnosis as exclusion criteria and potentially even larger sample sizes, could help to validate our conclusions. Moreover, this study explored genetic variants associated with factors extracted from a validated ALT questionnaire. Our findings of genetic diversity between ALTs should therefore encourage and seek for replication in future ALT research relying on questionnaires developed for each of these ALTs.

Our PRS-based analyses indicate a significant genetic association between polygenic risk scores for ASDs and ‘rigidity’; however, ASD-related variants could explain at most 0.20% of the genetic variance for ‘rigidity’. This result differs from previous a report that demonstrated a genetic correlation between population-based autistic traits and autistic formal diagnoses of ~0.50–0.60 (Colvert et al., 2015). The polygenic nature of ALTs, the phenotypic variability in our cohorts and the difference between base and target sample sizes could potentially account for the low variance explained in this study. However, our population-based genetic analyses investigate specifically common genetic variants, while previous twin studies also included rare variants and gene × environment interactions that might contribute to the observed difference in findings. However, our results seems consistent with previous reports applying the PRS-based method to neuropsychiatric phenotypes. Namely, PRS for OCD explained only 0.2% of the variance in obsessive-compulsive symptoms in the general population (den Braber et al., 2016). Also, although we observed significant association between PRS for ASDs and rigidity at different thresholds (*P_T_* = 0.05, 0.1 and 0.5), we could not find significant association when considering SNPs at the most conservative threshold (*P_T_* = 0.001). This result may indicate a combined effect of a wide range of small-effect ASD-related SNPs in predicting ALT variability. However, further PRS-based analyses, involving larger samples, are needed to clarify the genetic relationship between ASDs and ALTs. To further clarify the ALT-ASD genetic relationship, it would be valuable to assess the extent of symptoms variance explained by each ALT’s genetics specifically within individuals diagnosed with ASD. Large patient-based studies would be needed to address this point in future studies.

Finally, this study specifically investigated common genetic variations associated with population-based autistic traits. In light of the confirmed role of both common and rare variants in ASDs (De Rubeis et al., 2015; Grove et al., 2019; Iossifov et al., 2014), we believe that our understanding of the genetic architecture of ALTs could benefit from analysis of rare genetic variations, which should be considered as object of study in the future.

## Conclusion

Our analyses revealed genetic loci linked to ALTs in the general population which may be of relevance for ASDs. Our data demonstrate genetic concordance between ALTs and clinical ASDs demonstrating the potential to use population-based ALTs to address the complex ASD genetics. ALT-associated SNPs and genes seem involved in the immune response and eQTL signals for different ALTs are enriched for immune-related processes in the brain. These findings suggest an immune-ALT link that should inform further investigation. Overall, research on disorder-related traits has the potential to parse the heterogeneity of disorders and highlight dimension-specific biological pathway also important for pharmacotherapy.

## Supplemental Material

sj-pdf-1-aut-10.1177_13623613211019547 – Supplemental material for Potential role for immune-related genes in autism spectrum disorders: Evidence from genome-wide association meta-analysis of autistic traits Click here for additional data file. Supplemental material, sj-pdf-1-aut-10.1177_13623613211019547 for Potential role for immune-related genes in autism spectrum disorders: Evidence from genome-wide association meta-analysis of autistic traits by Martina Arenella, Gemma Cadby, Ward De Witte, Rachel M Jones, Andrew JO Whitehouse, Eric K Moses, Alex Fornito, Mark A Bellgrove, Ziarih Hawi, Beth Johnson, Jeggan Tiego, Jan K Buitelaar, Lambertus A Kiemeney, Geert Poelmans and Janita Bralten in Autism
