# Supplementary material for: Potential role for immune-related genes in autism spectrum disorders: Evidence from genome-wide association meta-analysis of autistic traits
Source: Autism. 2021 Aug 4;26(2):361–72. doi: 10.1177/13623613211019547 (PMC8814945; doi:10.1177/13623613211019547)
Supplement: sj-pdf-1-aut-10.1177_13623613211019547 – Supplemental material for Potential role for immune-related genes in autism spectrum disorders: Evidence from genome-wide association meta-analysis of autistic traits [file sj-pdf-1-aut-10.1177_13623613211019547.pdf]

Supplementary Information

Supplementary figures

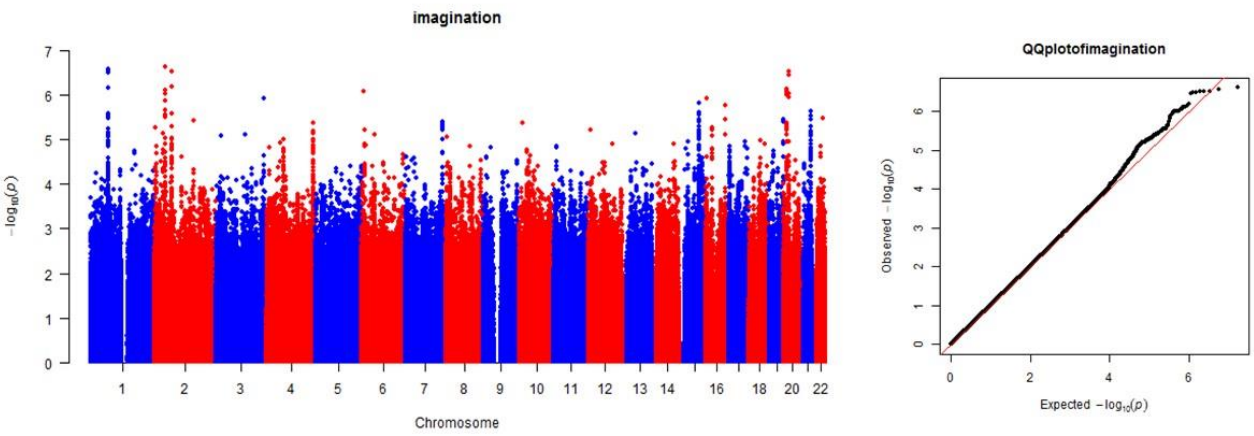

Figure 1. Manhattan and Q-Q plot of the GWAS meta-analysis for 'imagination'

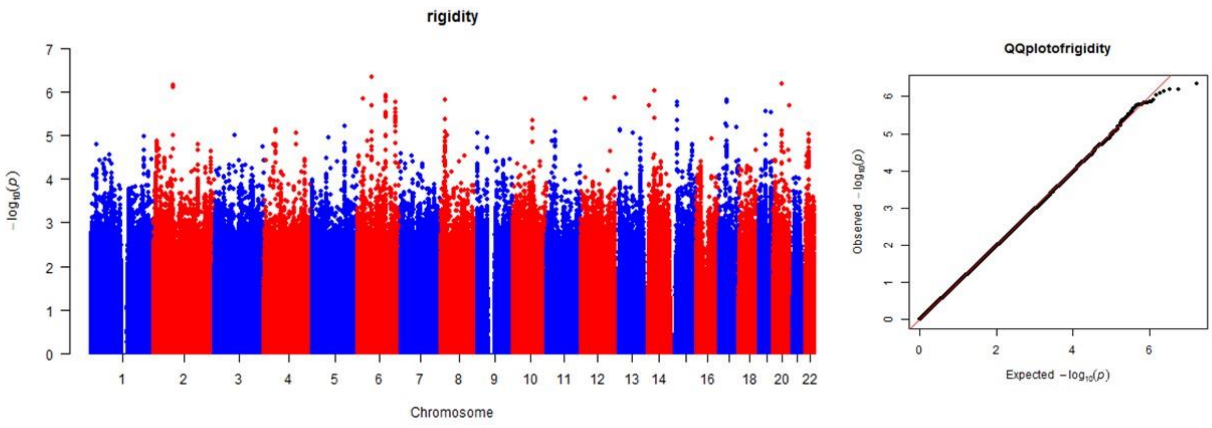

Figure 2. Manhattan and Q-Q plot of the GWAS meta-analysis for ' rigidity'

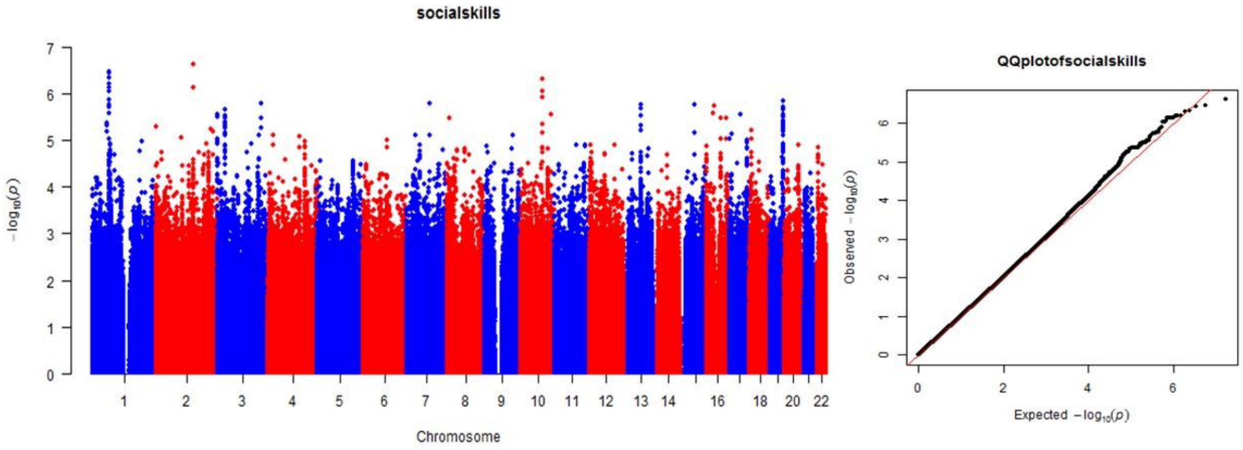

Figure 3 Manhattan and Q-Q plot of the GWAS meta-analysis for 'social skills'

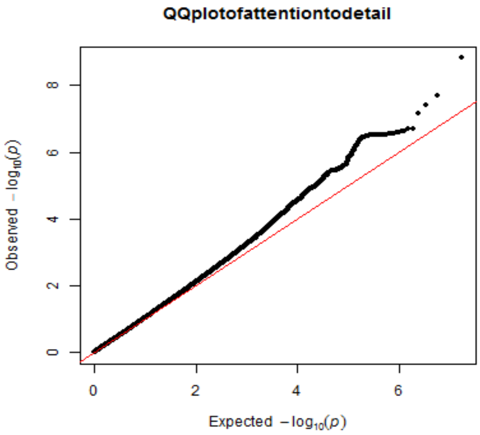

Figure 4 Q-Q plot for 'attention to detail'

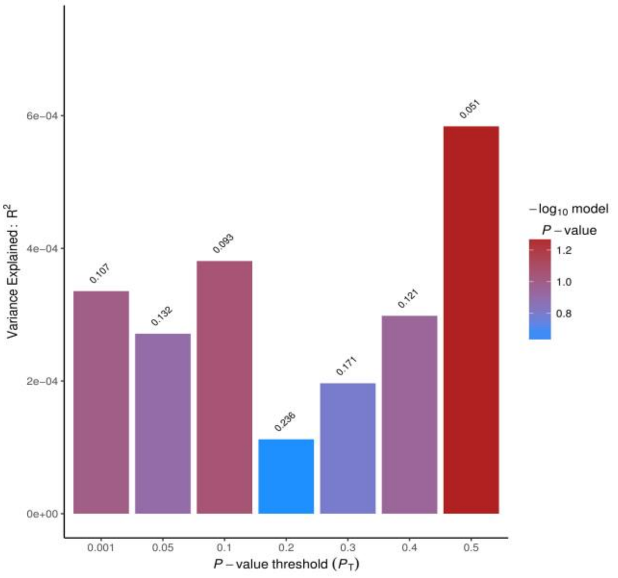

Figure 5 PRS-based results for ASD and 'attention to detail'

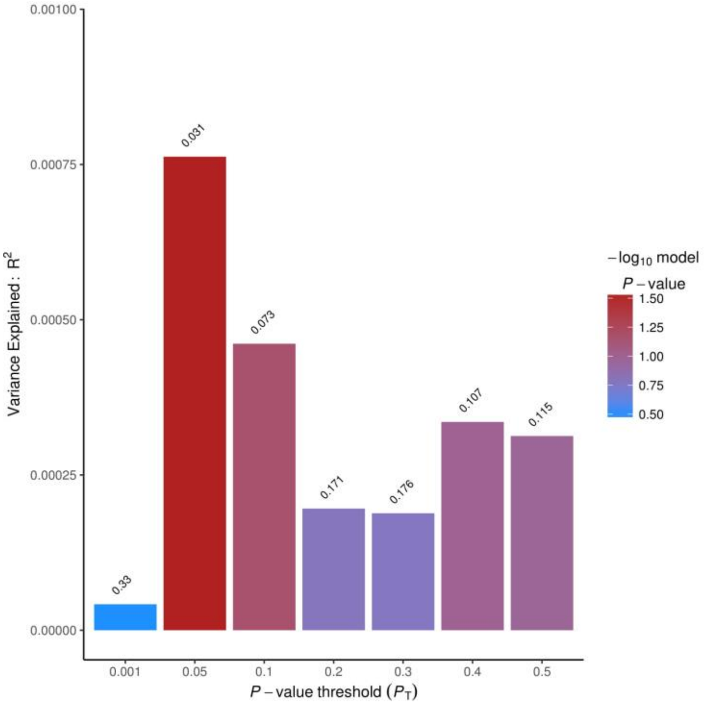

Figure 6 PRS-based results for ASD and 'imagination'

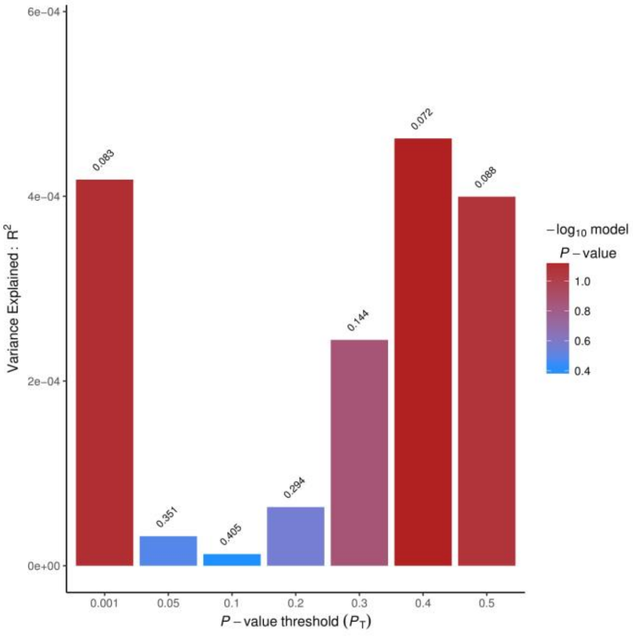

Figure 7 PRS-based results for ASD and ' social skills'

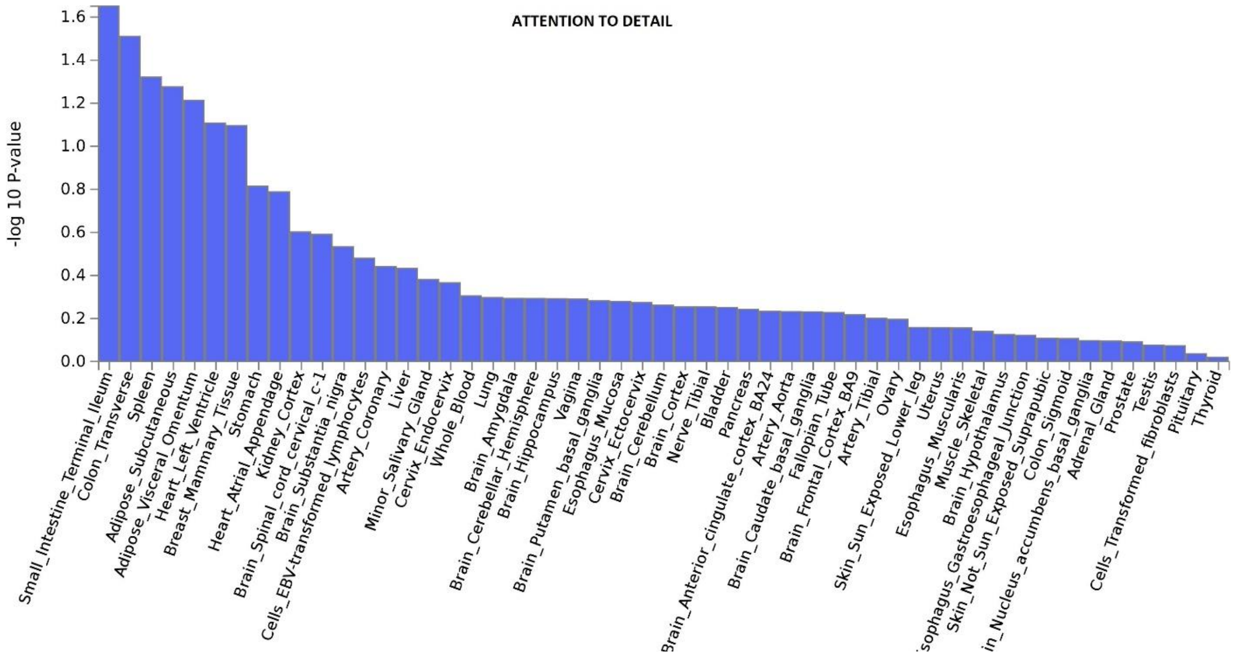

Figure 8 Gene expression linked to attention-to-detail across 53 GTEX-derived tissue types (FUMA-based analyses)

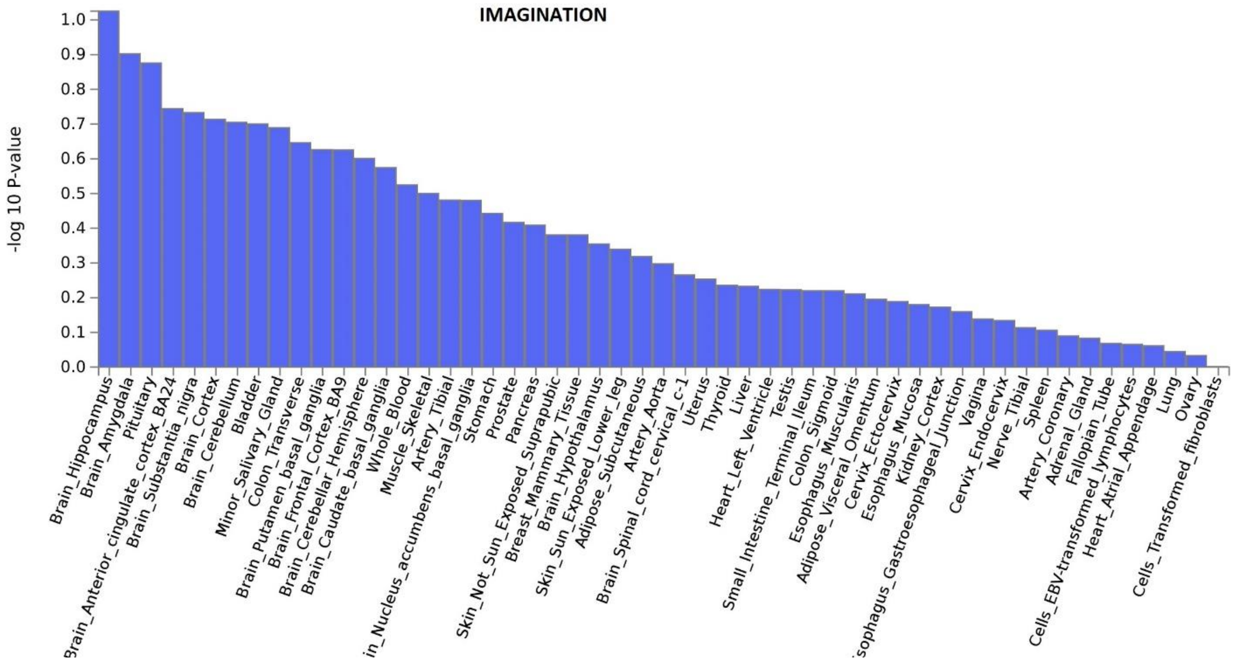

Figure 9 Gene expression linked to imagination across 53 GTEX-derived tissue types (FUMA-based analyses)

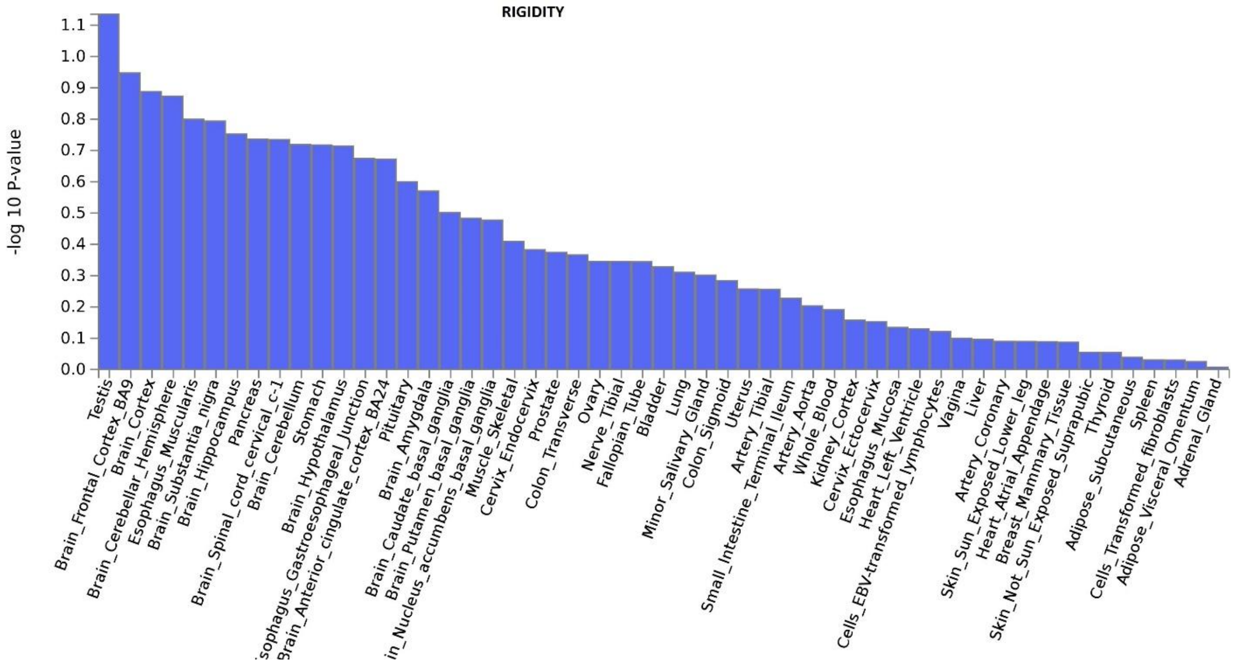

Figure 10 Gene expression linked to rigidity across 53 GTEX-derived tissue types (FUMA-based analyses)

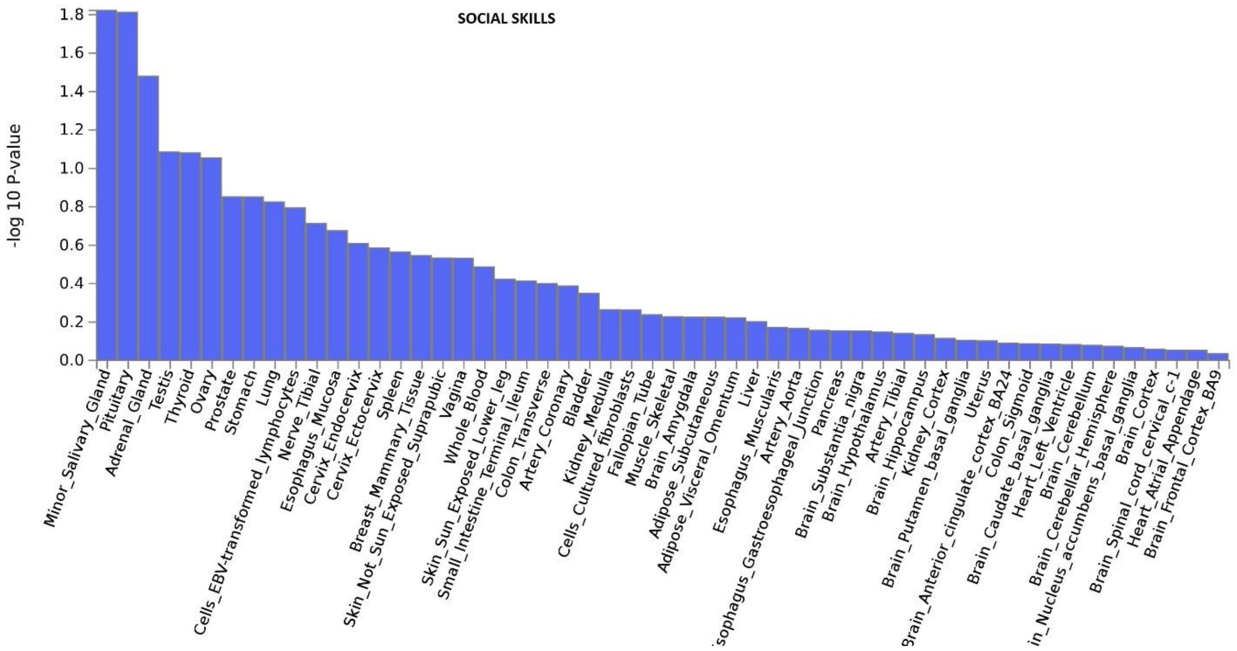

Figure 11 Gene expression linked to social skills across 53 GTEX-derived tissue types (FUMA-based analyses)

Supplementary Tables

Supplementary Table 1. Results of e-MAGMA-based gene co-expression network analyses for the 4 ALTs

Trait	Brain regions	Gene-set association p-value	Gene modules	Top ranked GO:BP
Attention	Putamen	0.01	salmon	Immune response
	Total Cortex	0.03	cyan	Response to cytokine
Imagination	Nucleus accumbens	0.003**	Dark red	Synaptic signalling
	Total Cortex	0.001**	black	neurogenesis

	amygdala	0.001**	Skyblue3	CNS development
Rigidity	Nucleus accumbens	0.01	red	Immune signalling
	Anterior cingulate cortex	0.01	lightcyan	Axon ensheatment
Social	Putamen	0.002**	midnightblue	Mitochondrial translation
	Anterior cingulate cortex	0.001**	magenta	RNA processing
	Frontal cortex	0.01	brown	Cellular metabolic process
	Amygdala	0.01	white	RNA metabolic process
Results of e-MAGMA gene co-expression network analyses. Reported are the significant ALT-specific association with gene modules across brain regions. Modules represent gene expression networks indexed by colour and referring to GO-based biological processes. CNS= central nervous system; GO= gene onthology; BP= biological processes. ** = significant results after Bonferroni-corrected *p = 0.007* (p-value divided by the number of region tested).				

Supplementary Table 2. Questions used to measure four autistic-like traits; a= reverse-scored item.

Questions used to measure four autistic-like traits
Attention to detail<br>*By looking at someone face, I find easy to work out what is he or she thinking or feeling*<br>*I can quickly workout whether someone is fascinated by what I say*<br>*I tend to notice details that others do not* $_a$<br><br>***Imagination***<br>*I find making stories up easy*<br>*As a child, I enjoyed playing games involving pretending with other children*<br><br>***Rigidity***<br>*People tell me that I keep going on and on about the same thing* $_a$<br>*I often get so absorbed that I lose sight of other things* $_a$<br>*It upsets me if my daily routine is disturbed* $_a$<br>*I prefer to do things the same way over and over again* $_a$

Social skills

I find it hard to make new friends [a]

I enjoy social occasions as birthdays, receptions, etc.

I don't know how to keep a conversation going [a]
